# A recombinant capripoxvirus expressing the F protein of peste des petits ruminants virus and the P12A3C of foot-and-mouth disease virus

**DOI:** 10.1186/s12917-022-03529-5

**Published:** 2023-01-21

**Authors:** Jidong Li, Jianlin Wang, Yanan Guo, Zhenxing Gong, Xuepeng Cai

**Affiliations:** 1 School of Agriculture, Ningxia University, Yinchuan, 750021 Ningxia China; 2 State Key Laboratory of Veterinary Etiological Biology, Lanzhou Veterinary Research Institute, Chinese Academy of Agricultural Sciences, Lanzhou, 730046 Gansu China; 3 Institute of Animal Science, Ningxia Academy of Agricultural and Forestry Sciences, Yinchuan, 750002 Ningxia China

**Keywords:** Goat pox virus, Peste des petits ruminants virus, Foot-and-mouth disease virus, P12A3C gene, F gene

## Abstract

**Background:**

Peste des petits ruminants (PPR), foot-and-mouth disease (FMD) and sheep pox and goat pox are three important infectious diseases that infect goats, sheep and other small ruminants. It is well-known that the prevention of three diseases rely mainly on their individual vaccines. However, the vaccines have a variety of different disadvantages, such as short duration of immunity, increasing the number of vaccinations, and poor thermal stability. The purpose of this study is to construct a recombinant goat pox virus (rGPV) capable of expressing the F gene of PPRV and the P12A3C gene of FMDV as a live vector vaccine.

**Results:**

The IRES, FMDV P12A3C and PPRV F genes into the multi-cloning site of the universal transfer plasmid pTKfpgigp to construct a recombinant transfer plasmid pTKfpgigpFiP12A3C, and transfected GPV-infected lamb testis (LT) cells with liposomes and produced by homologous recombination Recombinant GPV (rGPV/PPRVF-FMDVP12A3C, rGPV). The rGPV was screened and purified by green florescence protein (GFP) and xanthine-guanine-phosphoribosyltransferase gene (gpt) of *Escherichia coli* as selective markers, and the expression of rGPV in LT cells was detected by RT-PCR and immunofluorescence techniques. The results showed that the virus strain rGPV/PPRVF-FMDVP12A3C containing FMDV P12A3C and PPRV F genes was obtained. The exogenous genes FMDV P12A3C and PPRV F contained in rGPV were normally transcribed and translated in LT cells, and the expression products could specifically react with PPRV and FMDV antiserum. Then, the rGPV was intradermally inoculated with goats, the animal experiments showed that rGPV/PPRVF-FMDVP12A3C could induce high levels of specific antibodies against GPV, PPRV and FMDV.

**Conclusions:**

The constructed rGPV induced high levels of specific antibodies against GPV, PPRV and FMDV. The study provides a reference for “ one vaccine with multiple uses “ of GPV live vector vaccine.

**Supplementary Information:**

The online version contains supplementary material available at 10.1186/s12917-022-03529-5.

## Background

Peste des petits ruminants (PPR), foot-and-mouth disease (FMD), sheep pox and goat pox are major diseases that endanger goats, sheep and other small ruminants, which belong to Category A virulent infectious diseases issued by OIE. PPR, FMD, sheep pox and goat pox are prevalent worldwide and causing certain harm to global small ruminant industry [1–5].

Currently, the prevention of PPR, FMD, sheep pox and goat pox relies mainly on their individual vaccines. For example, the PPR vaccines are mainly two attenuated vaccines Nigeria/75/1 and Sungri/96 [6–8], the FMD vaccines are mainly inactivated vaccines of different serotypes [9–12], and the vaccines of sheep pox and goat pox are mainly vaccine strains formed from locally isolated wild viruses after weakening their virulence, such as China’s goat pox virus (GPV) AV41 used in prevention of sheep pox and goat pox [13, 14]. Because these three vaccines are all single vaccines, a series of immunization procedures need to be formulated in use. At the same time, the workload of immunization is also large, and the cost of immunization is relatively high. In order to solve the above problems, it is particularly important to develop a vaccine with multiple preventions in one shot.

A large number of existing studies have shown that goat pox virus is a good choice for the construction of live vector vaccines. The GPV genome is about 150 kb and is genetically stable. The coding region of thymidine kinase gene of GPV is a non-essential region for replication, which allows large capacity of exogenous gene insertion and expression. GPV and other pox viruses are being further studied as new vaccine vectors [14–18]. Peste des petits ruminants virus (PPRV) belongs to the genus Measles virus of the family Paramyxoviridae, and the fusion protein F on the surface of the viral capsule membrane is one of the main immunogens that PPRV induces neutralizing antibody formation [19–21]. Studies on foot-and-mouth disease virus (FMDV) have shown that P1 containing VP1, VP2, VP3, VP4 and 2A constitute the viral capsid and can therefore be used as antigenic genes for FMDV in related studies of recombinant vaccines [22, 23]. 3C of FMDV are responsible for cleavage of polyproteins to construct the viral capsid [22, 23]. Therefore, if the F gene of PPRV and the P12A3C gene of FMDV gene can be inserted into the GPV genome to construct a goat pox virus live vector vaccine, it will effectively solve the shortcomings of the existing vaccines and achieve the purpose of one shot and three defenses.

In this study, a universal transfer plasmid pTKfpgigp for GPV recombination was constructed. The F gene of PPRV and the P12A3C gene of O-type FMDV were inserted into this plasmid, and then the recombinant plasmid and GPV were co-transfected into LT cells. The rGPV carrying PPRV F gene and FMDV P12A3C gene was formed by homologous recombination. The purpose is to study rGPV as a candidate vaccine to achieve the purpose of multi-purpose and provide reference for the development of PPR and FMD recombinant marker vaccines.

## Results

### The PCR results of target genes

**Fig. 1:**
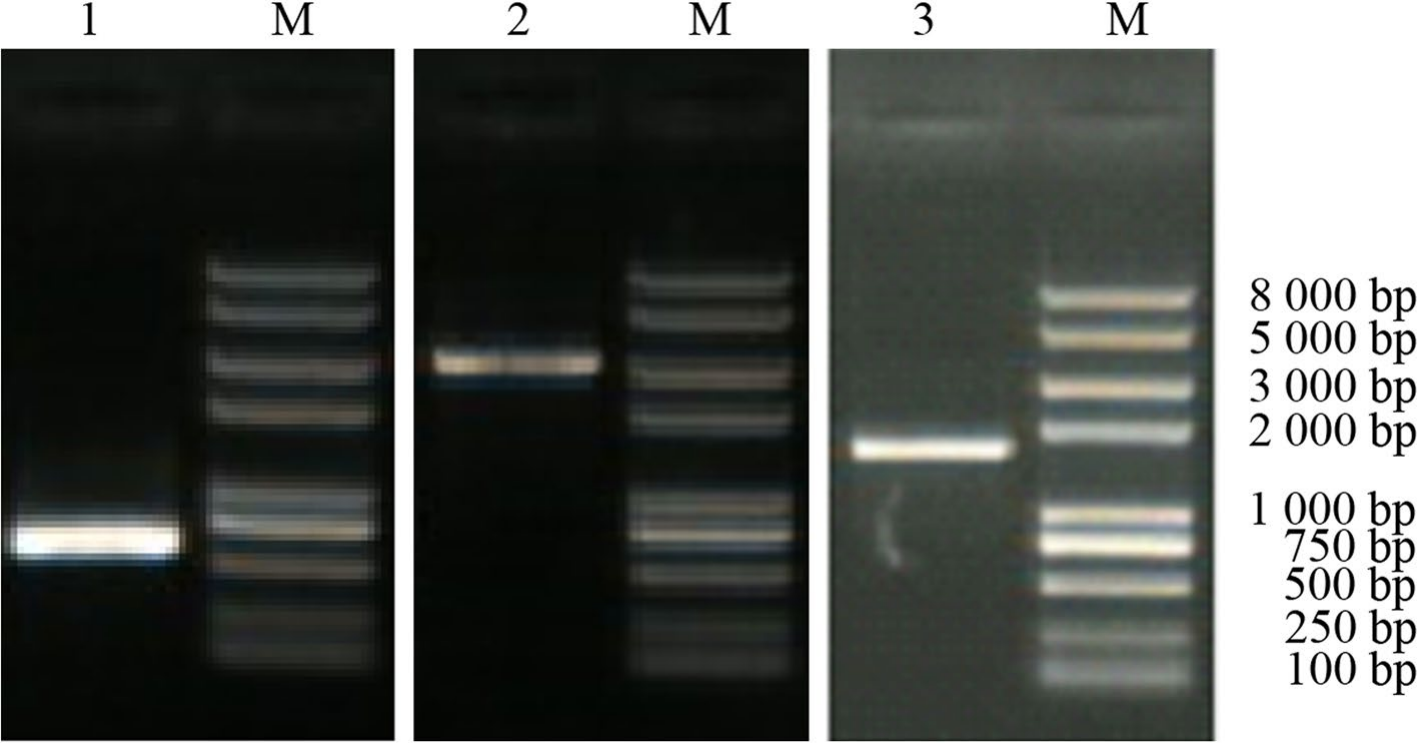
PCR amplification products of target genes. M Trans2K plus II DNA Marker. 1 IRES. 2 FMDV P12A3C. 3 PPRV F

IRES sequence, FMDV P12A3C and PPRV F gene were amplified by PCR. The IRES sequence was 606 bp, the P12A3C sequence was 2937 bp, and the F gene was 1669 bp (Fig. 1).

### Construction and identification of recombinant plasmid

**Fig. 2:**
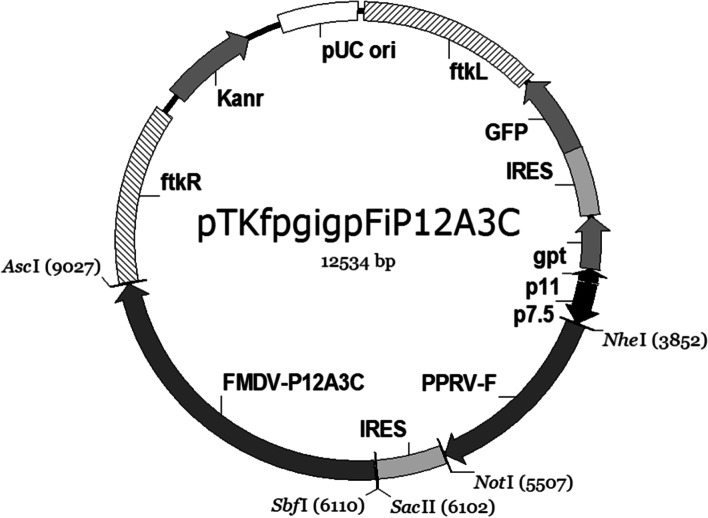
The schematic of the recombinant plasmids construction

**Fig. 3:**
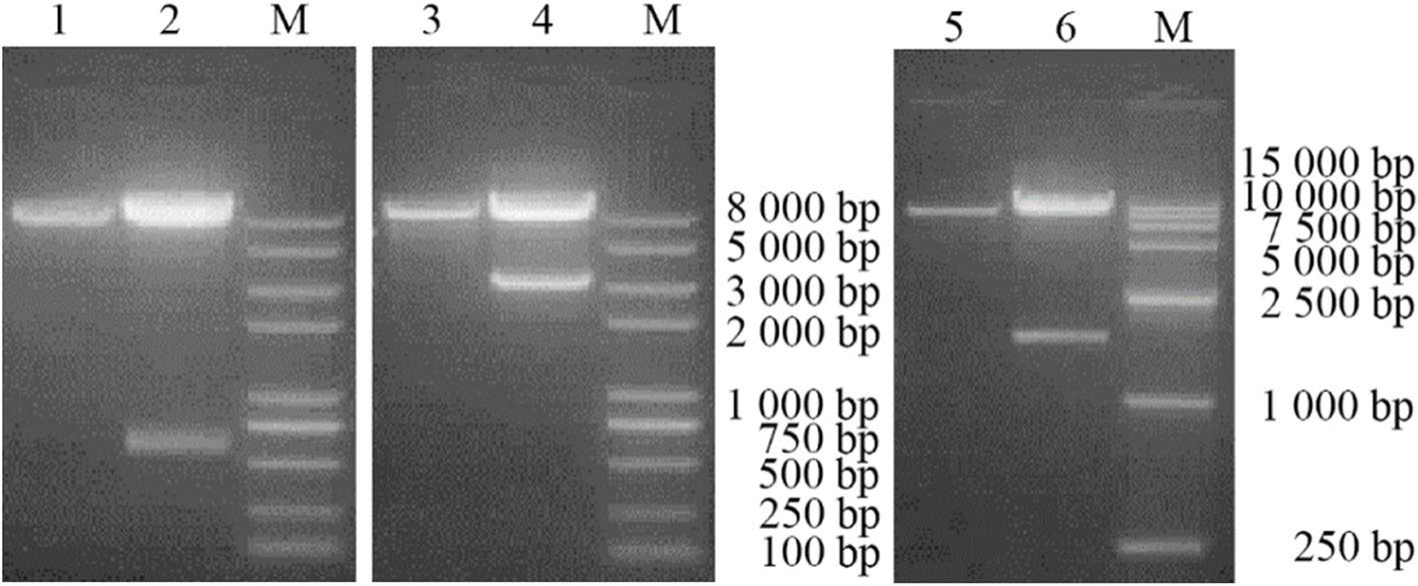
Identification of recombinant plasmid pTKfpgigpi、pTKfpgigpiP12A3C、pTKfpgigpFiP12A3C by restriction enzyme digestion. M Trans2K plus II DNA Marker. 1 Product from the plasmid pTKfpgigp digested with *Not*Iand *Sac*II. 2 Product from the plasmid pTKfpgigpi digested with NotI and *Sac*II. 3 Product from the plasmid pTKfpgigpi digested with *Sbf*I and *Asc*I. 4 Product from the plasmid pTKfpgigpiP12A3C digested with *Sbf*I and *Asc*I. 5 Product from the plasmid pTKfpgigpi-P12A3C digested with *Nhe*I and *Not*I. 6 Product from the plasmid pTKfpgigp-F-iP12A3C digested with *Nhe*I and *Not*I

The tranfer plasmid was designed as pTKfpgigpFiP12A3C (Fig. 2). The recombinant plasmids pTKfpgigpi, pTKfpgigpiP12A3C and pTKfpgigpFiP12A3C were identified by double digestion with Not I and Sac II, Sbf I and Asc I, Nhe I and Not I, respectively (Fig. 3).

### The formation and screening results of rGPV

**Fig. 4:**
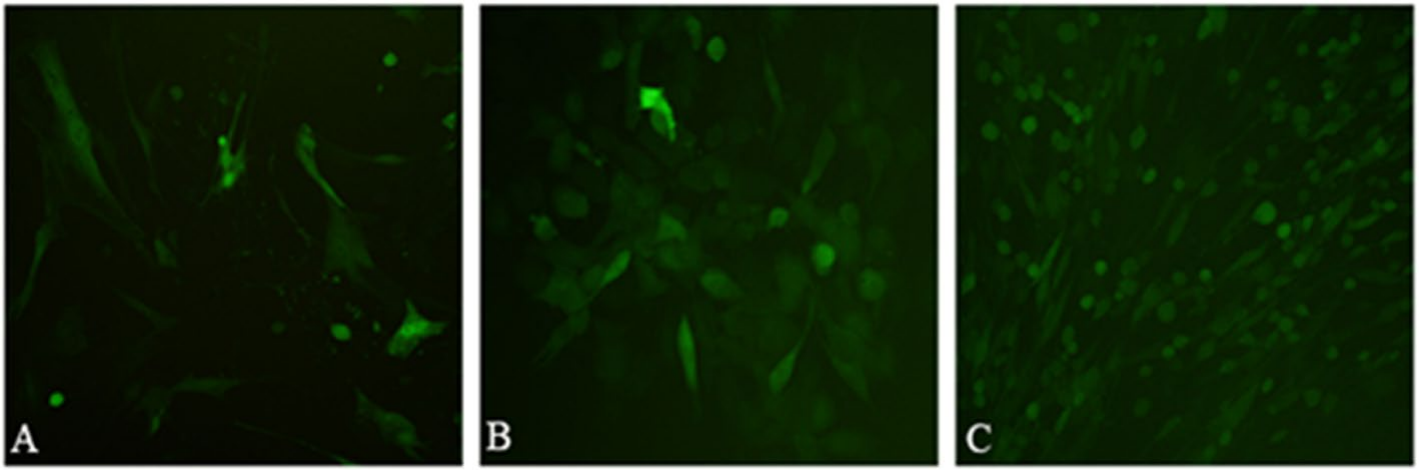
Green fluorescence during the formation and selection of rGPV. **A** green fluorescence in the LT cells transfected into the plasmids pTKfpgigpFiP12A3C. **B** single green fluorescence plague in the LT cells infected rGPV. **C** green fluorescence CPE in the LT cells infected rGPV

The recombinant plasmid pTKfpgigpFiP12A3C was transfected into LT cells infected with GPV AV41. The green fluorescence was observed under fluorescence microscope at 24 h after transfection, and the fluorescence peaked at 72 h, indicating that the reporter gene was normally expressed. At the beginning of rGPV screening, the distribution of lesions with green fluorescence. When the positive stain was continuously transmitted to the 4th to 5th generations, a large number of CPE with green fluorescence could be observed (Fig. 4).

### RT-PCR results of exogenous gene of rGPV

**Fig. 5:**
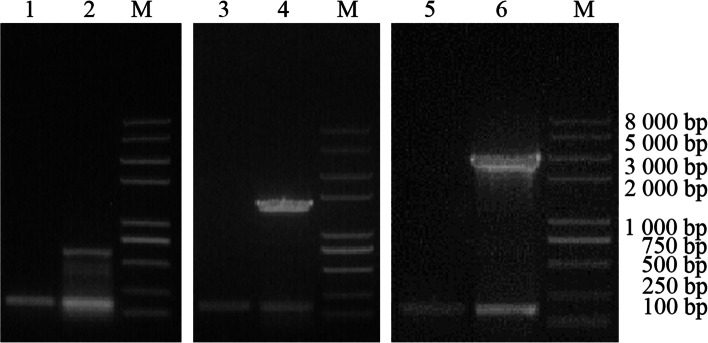
Identification of the exogenous gene in rGPV by RT-PCR. RNA from the GPV-infected LT cells. M Trans2K plus II DNA Marker. 1, 3, 5 β-actin (160 bp). 2 IRES sequence (606 bp). 4 PPRV-F sequence (1669 bp). 6 FMDV-P12A3C sequence (2937 bp)

The transcription of IRES, PPRV F and FMDV P12A3C in rGPV was detected by RT-PCR, which is consistent with the expectation (Fig. 5).

### Detection results of exogenous gene expression products in rGPV

**Fig. 6:**
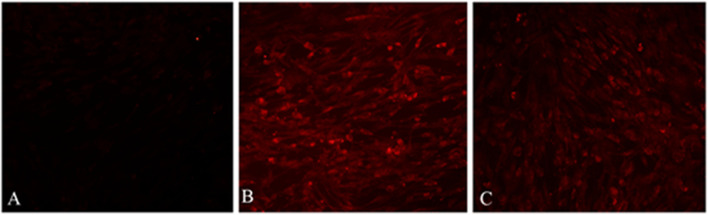
Immunofluorescence detection of exogenous protein in rGPV-infected LT cells. **A** LT cells infected with GPV. **B** LT cells infected with rGPV and reacted with PPRV antiserum. **C** LT cells infected with rGPV and reacted with FMDV antiserum

The expression products of PPRV F gene and FMDV P12A3C gene of rGPV in LT cells were detected by immunofluorescence with PPRV rabbit anti-positive serum and FMDV mouse anti-positive serum as primary antibody, and the goat anti-rabbit and goat anti-mouse TRITC-IgG as secondary antibody. The results showed that after rGPV infection in LT cells for 48 h, bright red fluorescence could be observed by indirect immunofluorescence (IIF) method, indicating that the exogenous gene carried by rGPV was normally expressed (Fig. 6).

### Immunogencity of rGPV

**Fig. 7:**
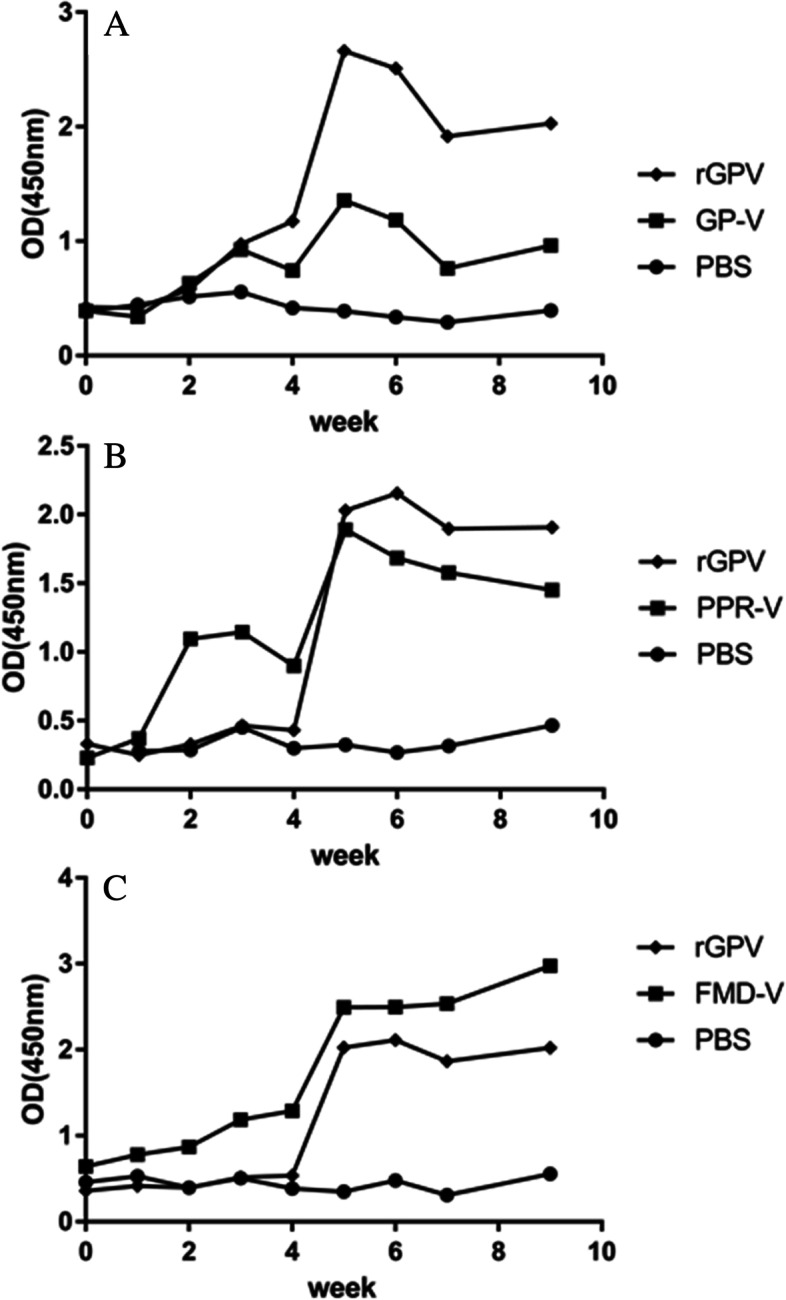
Detection levels of antibody. **A** anti-GPV. **B** anti-PPRV. **C** anti-FMDV

**Fig. 8:**
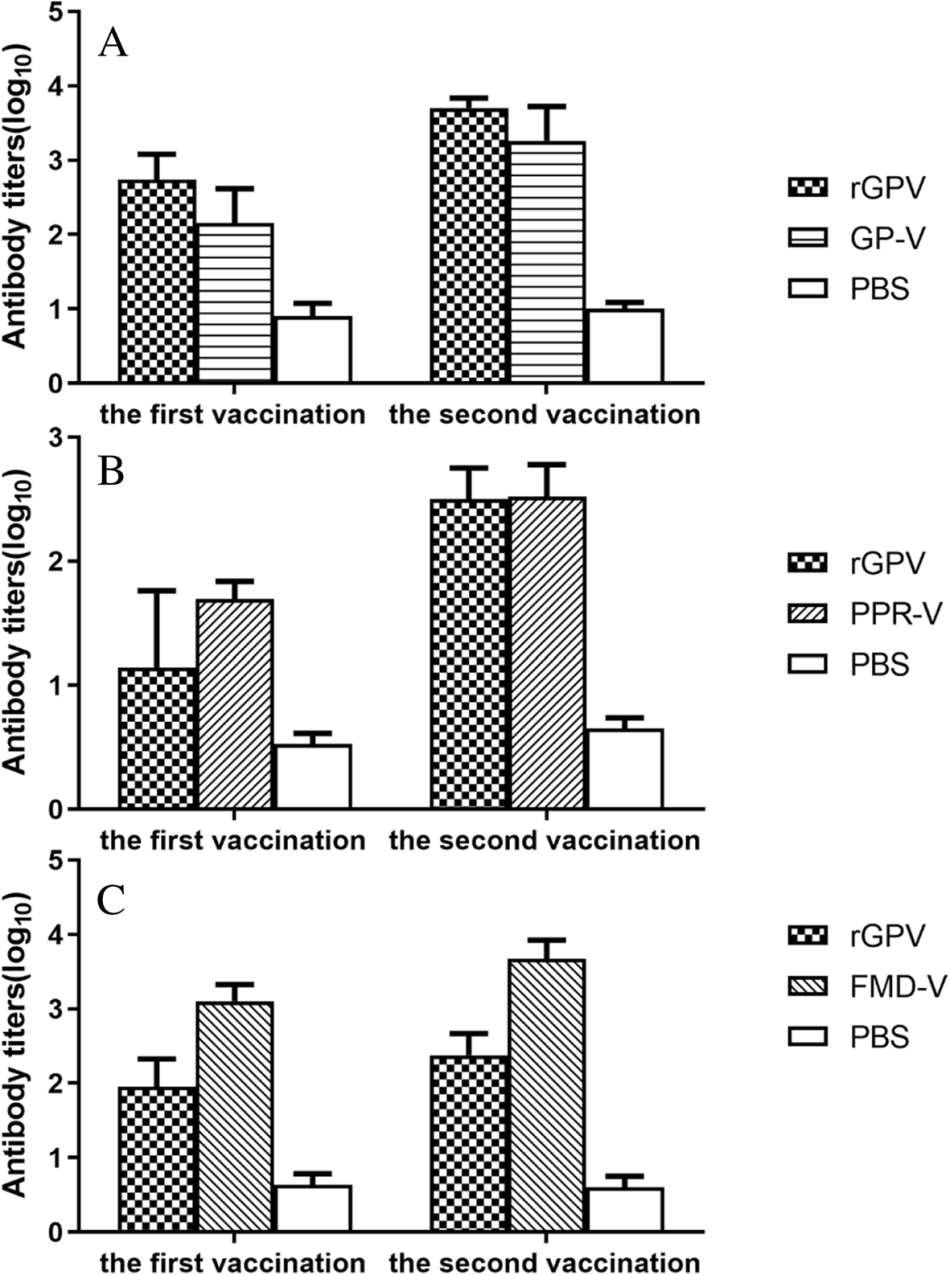
Comparison of levels of antibody. **A** anti-GPV. **B** anti-PPRV. **C** anti-FMDV

The serums were collected weekly before and after immunization for 9 times. And then the serums were diluted by 1:32 and the levels of anti-GP, PPR and FMD antibodies were detected using indirect ELISA test kit for antibodies to GPV, PPRV and FMDV respectively. After the first immunization, the antibody levels of each experimental group increased slowly. After strengthening immunization, the antibody levels in each group reached the peak within 1–2 weeks (Fig. 7). Comparing antibody titers of the 4th and 6th week serums respectively, the rGPV group was higher than the GP vaccine group, but the difference was not significant (*p* > 0.05) (Fig. 8A). The PPR antibody level showed different conditions. From the measured results, the anti-PPR antibody level was in an undetectable state after the first immunization with rGPV, and increased rapidly after booster immunization, with no significant difference from PPR-V in the vaccine control group (Fig. 8B). The results of FMD antibody detection showed that the FMD-V of the vaccine group was always higher than that of the rGPV group after the first immunization or enhanced immunization, and there was significant difference between the two groups (*p* < 0.01) (Fig. 8C).

## Discussion

The use of rGPV as a live vector vaccine has received the attention of many researchers. Different research groups have constructed rGPV strains containing different pathogen antigen genes and used them as immunogens for animal experiments. The results have confirmed that rGPV was able to induce antibody responses to the encoded antigens making it a good vaccine candidate [14, 18, 24–28]. However, these recombinant GPVs had expressed only one exogenous antigen gene. In this study, we constructed the rGPV expressing two antigen genes derived from PPRV and FMDV respectively, expanding the application of rGPV as vaccine. The PPRV F gene and FMDV P12A3C gene of rGPV were expressed normally in LT cells. Analyzing the research results, the reason may be that the immunization dose of the vaccine group was 1 dose, its TCID_50_ was 10^3.5^, and it was lower than that of the rGPV group. After the first inoculation, rGPV was unable to detect anti-PPR and FMD antibodies. The reason may be that many viral vectors require greater than a single immunization to induce immunity to the vectored antigen. In addition, the coating antigen used to detect the antibody level is PPRV inactivated whole virus, which may be related to the inability of anti-PR antibody to be detected after the first immunization.

Due to the deletion of *tk* gene, the proliferation rate of rGPV in LT cells was slower, and the time for CPE production was prolonged. Therefore, in the process of screening rGPV, it was difficult to isolate recombinant virus and parental virus. In the study of recombinant poxviruses, the *tk* gene of the virus itself was once used as a selection marker, and later reporters such as LacZ and GFP were used as selection markers [29–32]. In addition, referring to the screening of stable transfection in mammalian cells, researchers introduced *gpt* gene in the construction of vaccinia virus vector, which was similar to the selection effect of *neo* gene in mammalian cells, making the screening of recombinant viruses more time-saving and labor-saving [33]. In this study, after referring to the results of such studies, *gpt* gene was introduced as a pressure gene in the screening system, and GFP was used as a reporter gene to optimize the screening and purification process of rGPV strains. The results showed that this strategy quickly obtained recombinants of goat pox virus, making the construction of rGPV convenient and fast.

In the past, rGPV was constructed by using the genome of GPV to copy the restriction enzyme sites in the non-essential region, the exogenous gene was inserted into this site, and the transfer plasmid was constructed. Then the recombinant transfer plasmid and GPV parental strain were co-transfected into cells for homologous recombination [14–16]. In this case, if different exogenous genes need to be replaced to produce different GPV recombinant strains, recombinant design and construction of transfer plasmids are required, which increases the difficulty of research and prolongs the working time. In this study, a universal transfer plasmid pTKfpgigp for GPV recombination was constructed, which prolonged the homologous arm sequence of the recombination compared with the existing findings, and *gpt* and GFP double markers were selected as the screening system. In addition, a multiple cloning site was introduced downstream of the promoter p7.5, which could conveniently insert the desired exogenous gene. The experimental results showed that the design brought great convenience to the construction of rGPV.

In this study, the PPRV antigen gene F and FMDV P12A3C genes were inserted into the recombinant universal transfer plasmid pTKfpgigp of goatpox virus, and the rGPV containing two viral antigen genes was successfully constructed by homologous recombination and the immune effect was verified. It will be explored for rGPV as a vaccine candidate to achieve the purpose of multi-use of one vaccine.

## Conclusion

In conclusion, a recombinant capripoxvirus expressing the F protein of PPRV and the P12A3C of FMDV were successfully constructed in this study, and the recombinant GPV can significantly improve the antibody level of PPR, FMD, and GP. It is expected to become a new recombinant live vector vaccine.

## Methods

### Recombinant plasmid design

The plasmid pTKfpgigp was designed and constructed by the State Key Laboratory of Veterinary Etiological Biology, Lanzhou Veterinary Research Institute, Chinese Academy of Agricultural Sciences. This plasmid contains homology arms consistent with the goat pox virus *tk* gene and flanking sequences (ftkL, ftkR), the vaccinia virus promoters p11 and p7 .5 which was linked in reverse form, and the *gpt* and *gfp* genes were linked downstream of p11 as stress screening genes and reporter genes [34] (Fig. 2). On this basis, the F gene of PPRV and the P12A3C gene of FMDV were inserted into the multiple cloning site downstream of the promoter p7.5 as exogenou antigen genes to construct the recombinant plasmid pTKfpgigpFiP12A3C (Fig. 2). The plasmid of internal ribosome entry site (IRES) sequence derived from the plasmid pIRES2-AcGFP1 (BD Bioscience-Clonetech).

### Amplification of target genes

The IRES target gene (585 bp) was amplified by PCR using the vector pIRES2-AcGFP1 as a template and using IRES-F and IRES-R. The P12A3C gene (2907 bp) was amplified by PCR using FMDV cDNA as templates with the primers of P1-F and 3C-R. The F gene (1648 bp) was amplified by PCR using PPRV cDNA as templates with the primers of PPRVF-F and PPRVF-R. Kozak sequence was introduced before the start codon ATG. The primers and the products were as follows in Table 1 (bold parts are restriction sites).

**Table 1:** The primers and the products

Product	Primers	Sequence	Product size	Tm
IRES	IRES-F IRES-R	AATT**GCGGCCGC**GCCCCTCTCCCTCC CCT**CCGCGG**TGTGGCCATATTATCATCG	585 bp	55 °C
FMDV P12A3C	P1-F 3C-R	TTT**CCTGCAGG**GCCACCATGGGAGCCGGACAATCCAGTCCGG TTTTT**GGCGCGCC**TTACTCGTGATGTGGTTCGGGGTCAATGT	2907 bp	52 °C
PPRV F	PPRVF-F PPRVF-R	TAA**GCTAGC**GCCACCATGGCACGGGTC TGGA**GCGGCCGC**CTACAGTGATCTCAC	1648 bp	55 °C
β-actin	ACT-F ACT-R	CCTTCAATTCCATCATGAA GCGATGATCTTGATCTTC	160 bp	55 °C

### Construction and identification of recombinant plasmids

The IRES fragment was digested with NotI and SacII and inserted into the multiple cloning site of pTKfpgigp to form a recombinant plasmid pTKfpgigpi. Next, the FMDV P12A3C gene was digested with SbfI and AscI, and the PPRV F gene was digested with NheI and NotI to inserte into the two sides of the IRES sequence in the plasmid pTKfpgigpi to construct a recombinant plasmid pTKfpgigpFiP12A3C. After the recombinant plasmid pTKfpgigpFiP12A3C was transformed into *E.coli* DH5α, it was incubated on LB + Kan plates and cultured at 37 °C for 12–14 h. A single colony was picked and inoculated into LB + Kan liquid and incubated with shaking at 37 °C, 200 r/min for 10–12 h. The plasmid pTKfpgigpFiP12A3C was extracted from screened *E. coli* DH5α culture by using EndoFree Plasmid Kit (QIAGEN, USA), and was identified by restriction enzyme digestion and DNA sequencing. Recombinant plasmids identified as positive were stored at − 20 °C.

### Co-transfection of LT cells with recombinant plasmid and GPV

The testes of sheep about 1 month old were aseptically harvested and the primary cells of lamb testis (LT) were isolated. When the LT cells were plated to 6-well cell culture plates and grew to 90%, the GPV vaccine strain was inoculated at a multiplicity of infection (MOI) of 0.1, and the cells were adsorbed at 37 °C for 2 h. After discarding the cell supernatant, the recombinant plasmid pTKfpgigpFiP12A3C was transfected into LT cells using the instructions of Lipofectamine™ 2000 Transfection Reagent Kit (Invitrogen, USA). After 48 h of transfection, the fluorescence was detected under the excitation condition of 489 nm by a fluorescence microscope, and the cytopathic effect (CPE) was observed at the same time. When 90% of cells developed CPE and green fluorescence appeared, the cells were incubated with selected culture medium (DMEM, containing 2.5% FBS, mycophenolic acid (MPA) 25 μg/mL, Xanthine 250 μg/mL, Hypoxanthine 15 μg/mL) for 16–24 h.

LT cells were plated on 96-well plates. After the cells grew into a monolayer, the selected culture medium was incubated for 16–24 h, and then the culture medium was discarded. The recombinant virus suspension obtained in the above test was freeze-thawed three times, diluted 10^2^ ~ 10^4^ times, and inoculated on LT cells incubated in selective culture medium in 96-well plate. After adsorption at 37 °C for 2 h, the selected culture medium was replaced for further culture. The CPE and fluorescence of the cells were observed. When the viral plaque with green fluorescence appeared, the virus plaque was picked and amplified. Via five rounds of plaque purification, the purified recombinant GPV (rGPV) was named rGPV/PPRVF-FMDVP12A3C (carrying PPRV F gene and FMDV P12A3C).

### Identification of exogenous gene expression of rGPV

LT cells were cultured in 25cm^2^ cell flasks to a monolayer, and the purified rGPV was inoculated at 1 MOI. The culture medium was discarded when the cells developed mild to moderate lesions. TRIzol Regeant (1 mL/10 cm^2^ culture area) was added to the cell flask, pipetted up and down to make cell detach and crack, and total RNA was extracted according to the TRIzol instructions. The obtained RNA was reverse transcribed (20 μL system) with the Revert Aid First Strand cDNA Synthesis Kit. PCR was performed with specific primers amplifying the P12A3C and F genes, along with an internal reference primer for sheep β-actin, ACT-F and ACT-R.

LT cells were plated into 24-well cell culture plates, and rGPV was inoculated at 0.1 MOI when the cells grew to 80 ~ 90% confluence, and GPV control wells and negative control wells were set up. When the cells developed mild lesions (about 72 hours), the culture medium was discarded, and the detection of P12A3C and F gene expression products was performed according to the operating procedure of immunofluorescence assay (IFA). The primary antibody was PPRV rabbit antibody-positive serum, FMDV murine antibody-positive serum (1:100), and the secondary antibodies were goat anti-rabbit and goat anti-mouse TRITC-IgG (1:200).

### Animal immunization assay

Twenty-five female goats aged 6–8 months from non-epidemic areas were selected and the blood was collected to detect antibodies to determine GPV, PPRV, and FMDV antibody negativity. They were randomly divided into groups of 5 animals each, housed in pens and provided hay, threshing corn and clean water. Daily observations and physical examinations were performed during the trial to assess the health of the animals. The first group received rGPV, the second group received GP live attenuated vaccine, the third group received FMD inactivated vaccine, the fourth group received PPR live attenuated vaccine, and the fifth group was the PBS control group. The inoculation method was as follows: rGPV was intradermally injected into the tail of goat at 10^5^ TCID_50_, GP vaccine was intradermally injected into the tail of goat, FMD vaccine was intramuscularly injected into goat, PPR vaccine was intramuscularly injected into goat, and PBS was intramuscularly injected. Booster immunization was performed 4 weeks after the first immunization at the same dose and by the same immunization route. Serums were collected once before the test and once a week after the first immunization and after the second immunization. Serums were separated and antibody levels were measured by indirect ELISA. Anti-GPV antibodies were detected using indirect ELISA kit for detecting antibodies of sheep pox and goat pox virus (Biovetest， China). Anti-FMDV antibodies were detected using indirect ELISA kit for detecting antibodies of foot and mouth disease virus (Kanglang, China). Anti-PPRV antibodies were detected using indirect ELISA kit for detecting antibodies of peste des petits ruminants virus (Enzyme-linked, China). All test were performed as the product instructions.

## Supplementary Information

**Additional file 1.**

## Data Availability

All data generated or analyzed during this study are included in this article.

## Abbreviations

PPR: Peste des petits ruminants
PPRV: Peste des petits ruminants virus
FMD: Foot-and-mouth disease
FMDV: Foot-and-mouth disease virus
GPV: Goat pox virus
rGPV: Recombinant goat pox virus
IRES: Internal ribosome entry site
GFP: Green florescence protein
gpt: Xanthine-guanine-phosphoribosyltransferase
LT: Lamb testis
MOI: Multiplicity of infection
MPA: Mycophenolic acid
CPE: Cytopathic effect
IFA: Immunofluorescence assay
IIF: Indirect immunofluorescence
