# Supplementary figures and images for: A recombinant capripoxvirus expressing the F protein of peste des petits ruminants virus and the P12A3C of foot-and-mouth disease virus
Source: BMC Vet Res. 2023 Jan 21;19:18. doi: 10.1186/s12917-022-03529-5 (PMC9863095; doi:10.1186/s12917-022-03529-5)

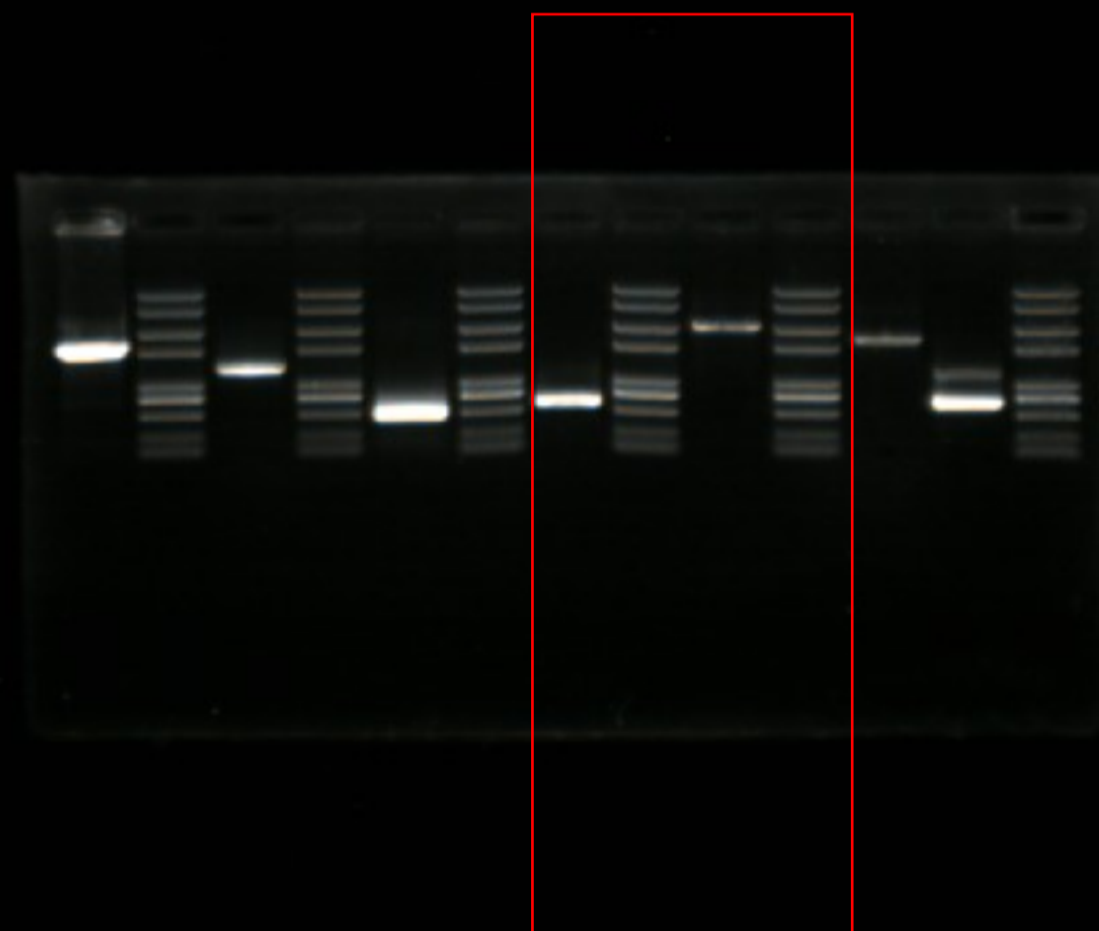

Fig. 1

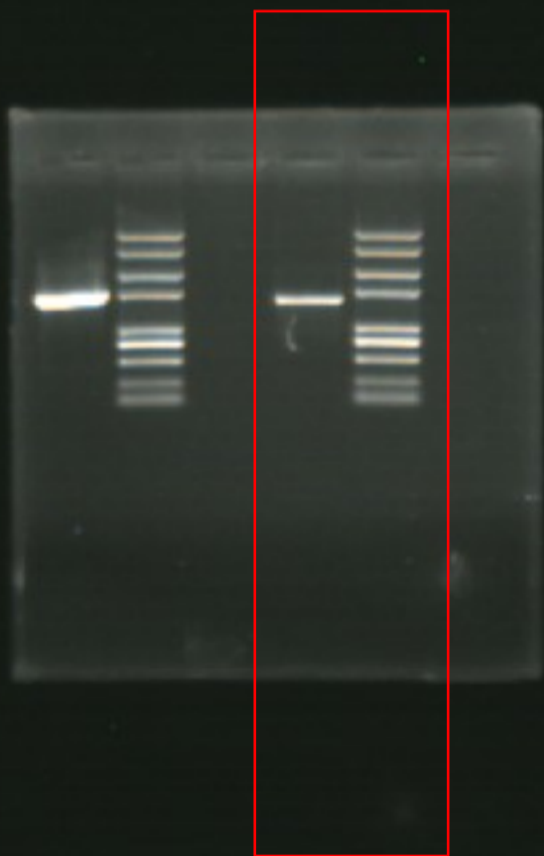

Fig. 1

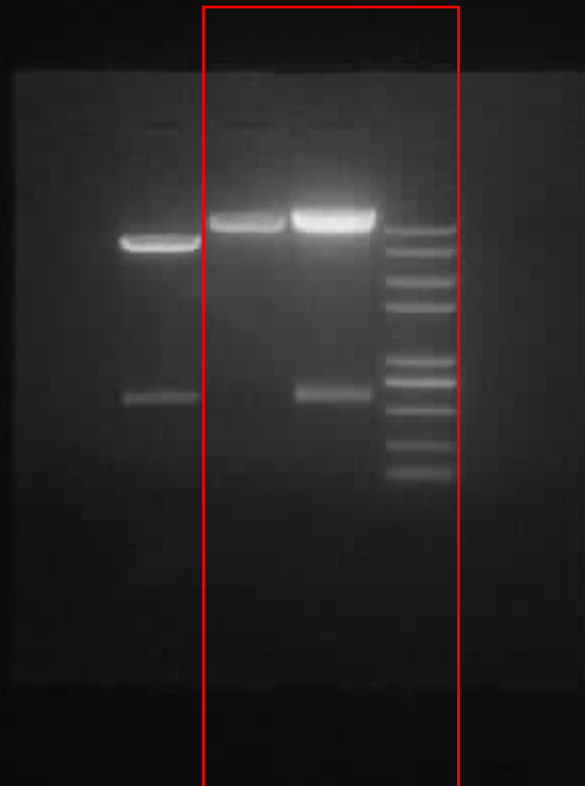

Fig. 3

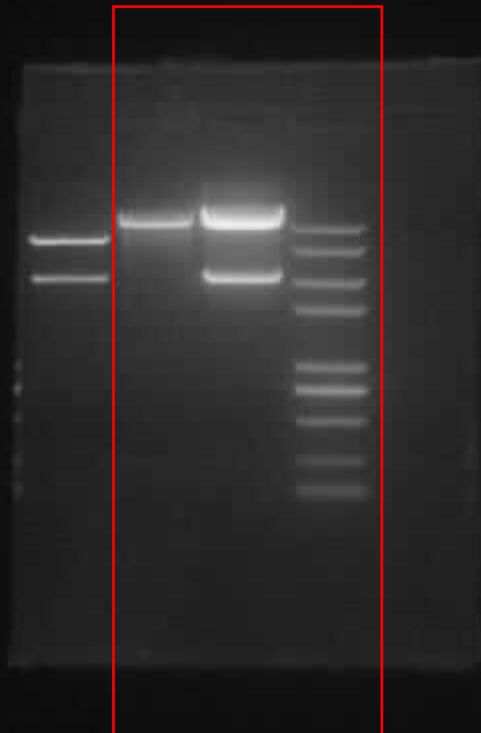

Fig. 3

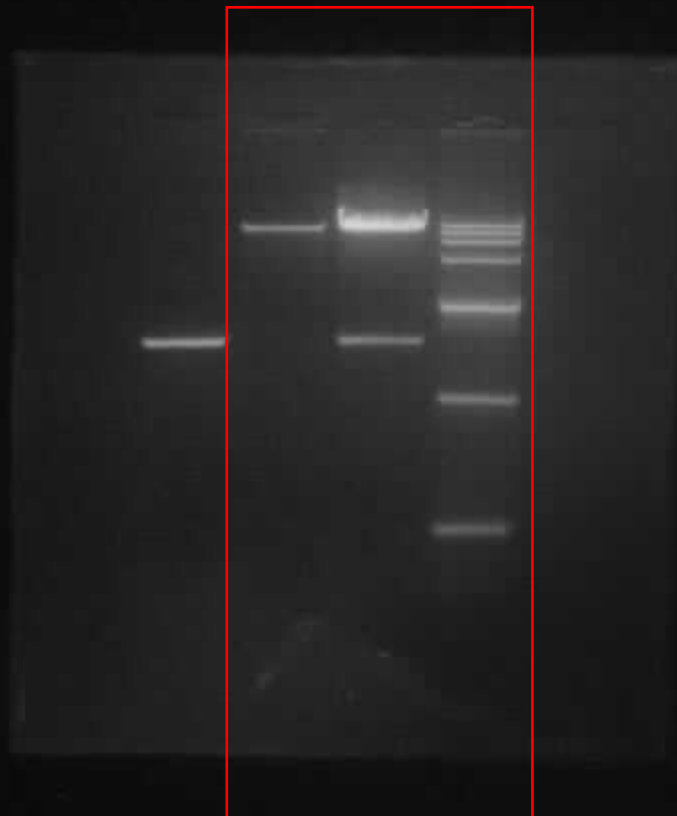

Fig. 3

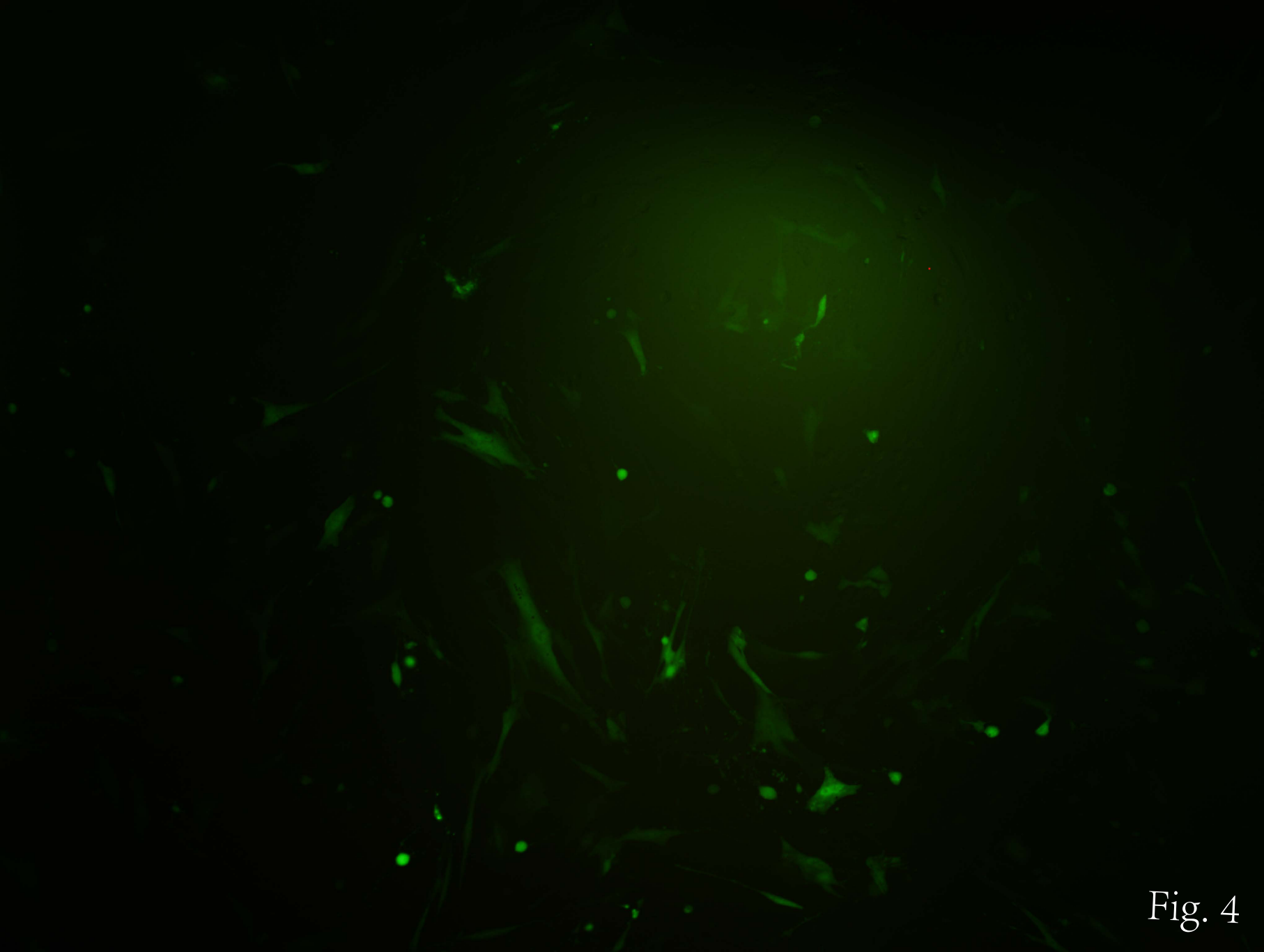

Fig. 4

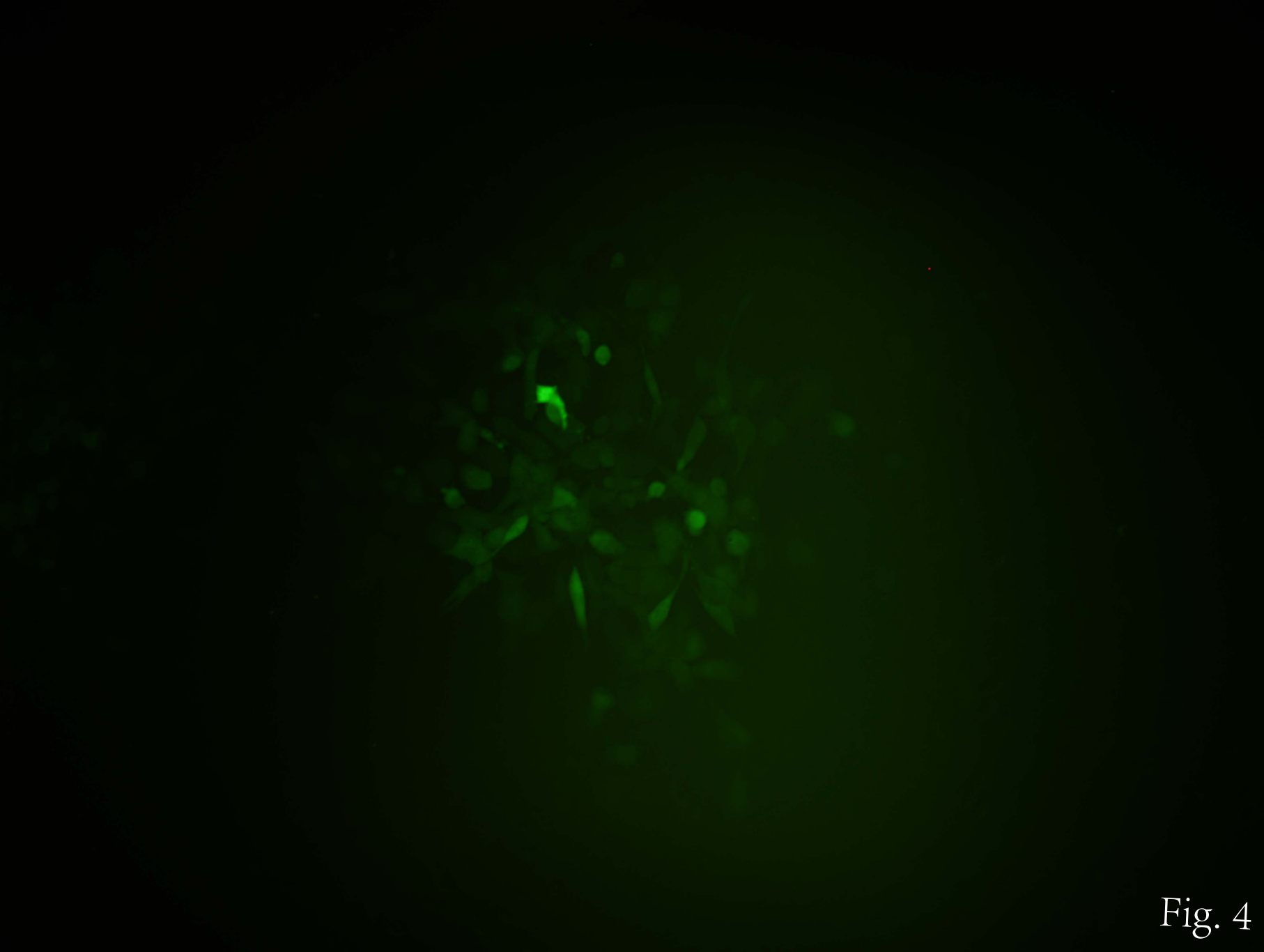

Fig. 4

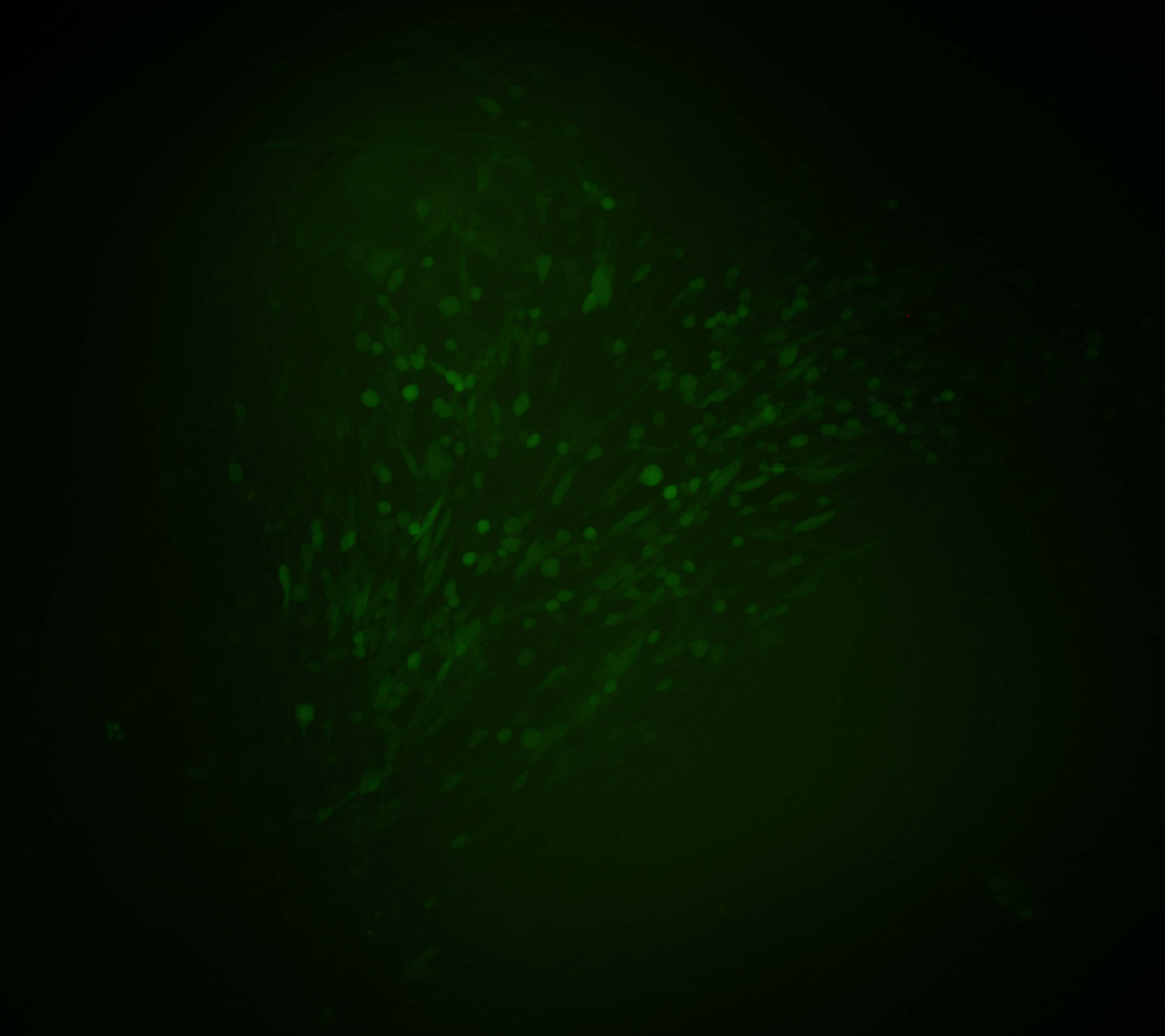

Fig. 4

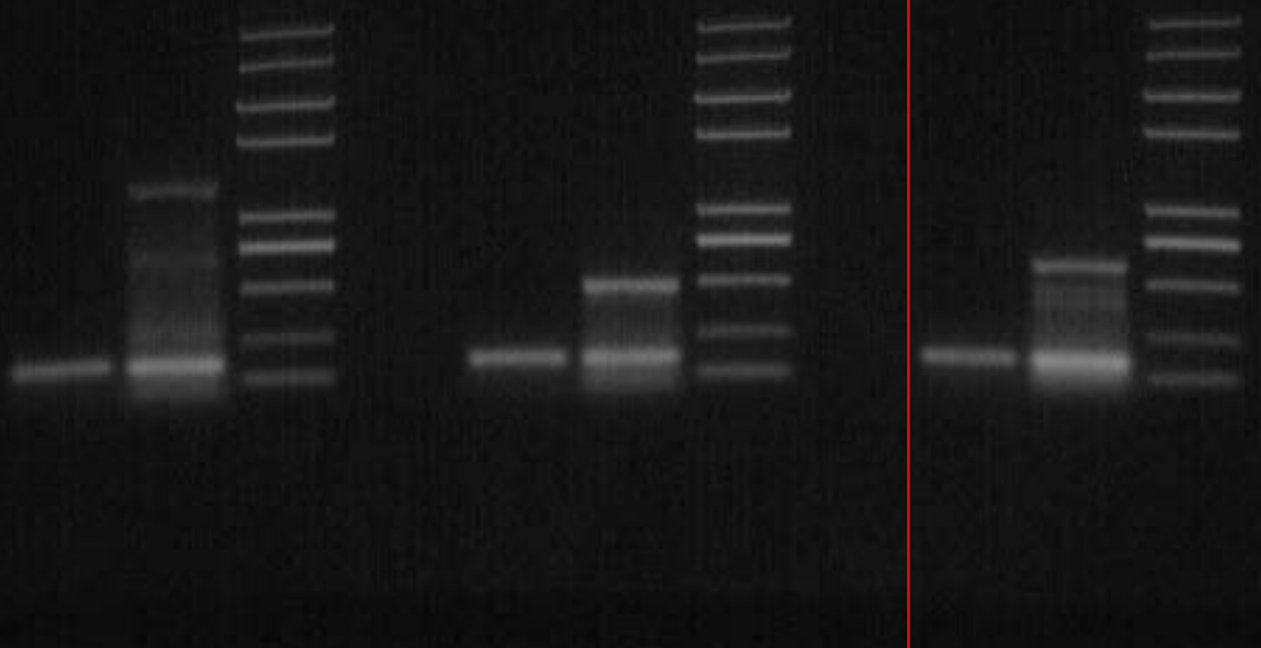

Fig. 5

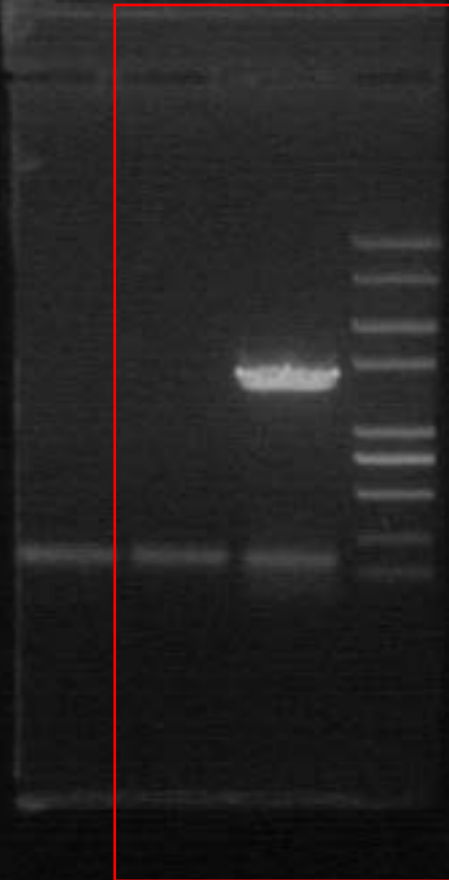

Fig. 5

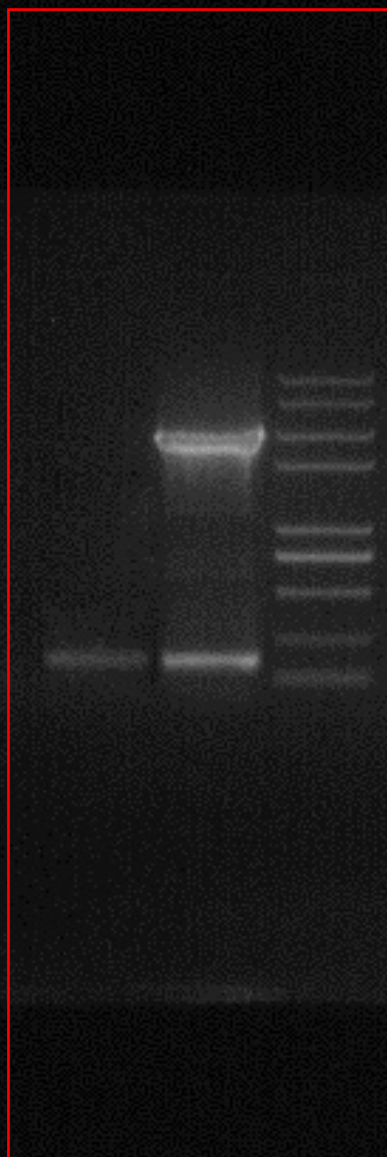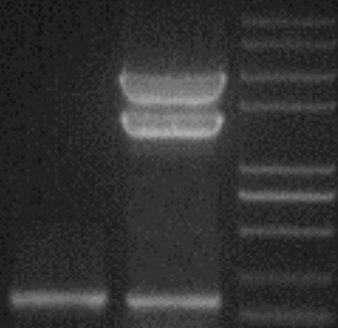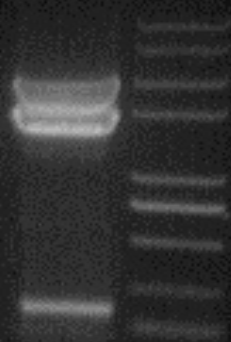

Fig. 5

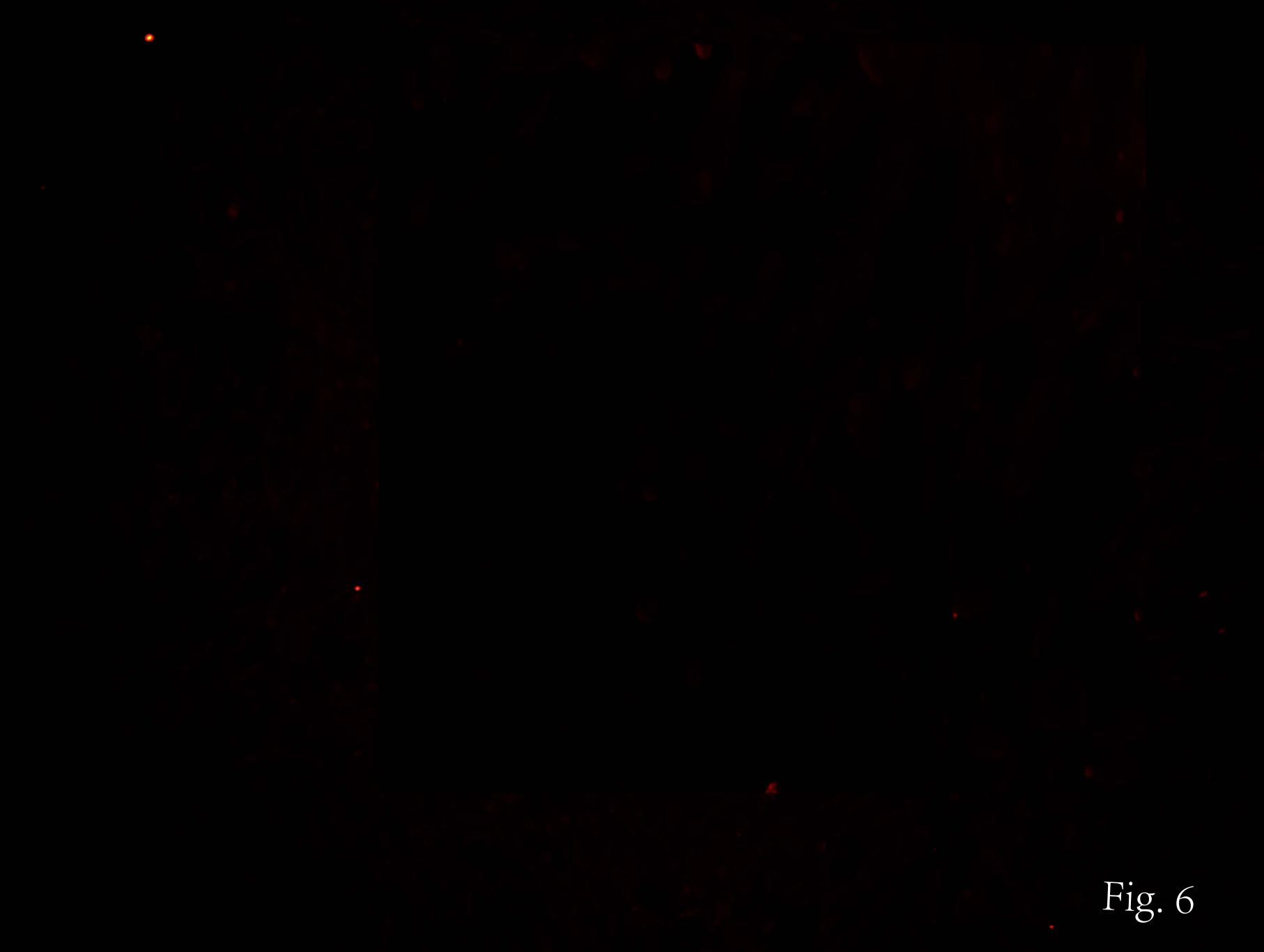

Fig. 6

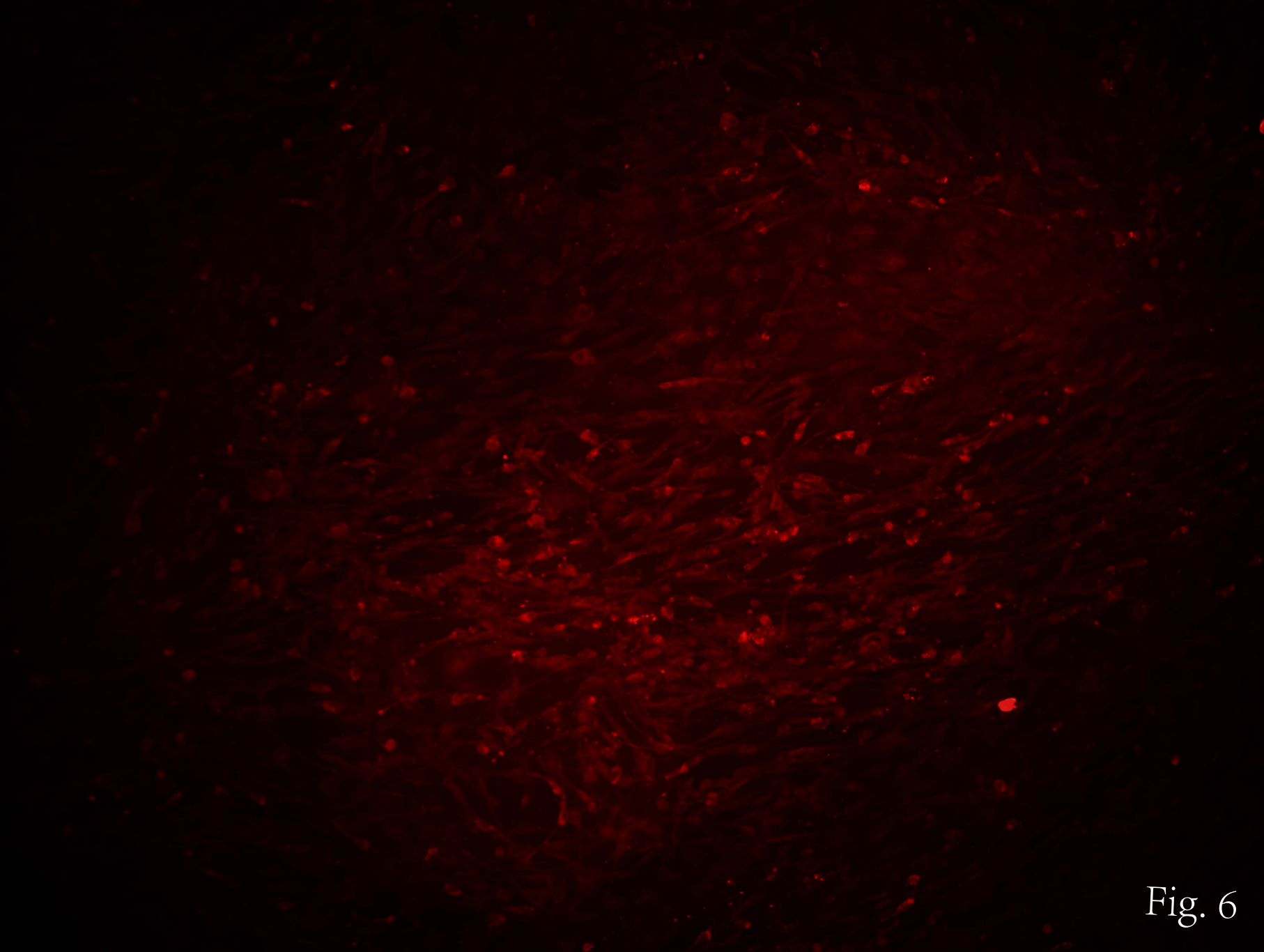

Fig. 6

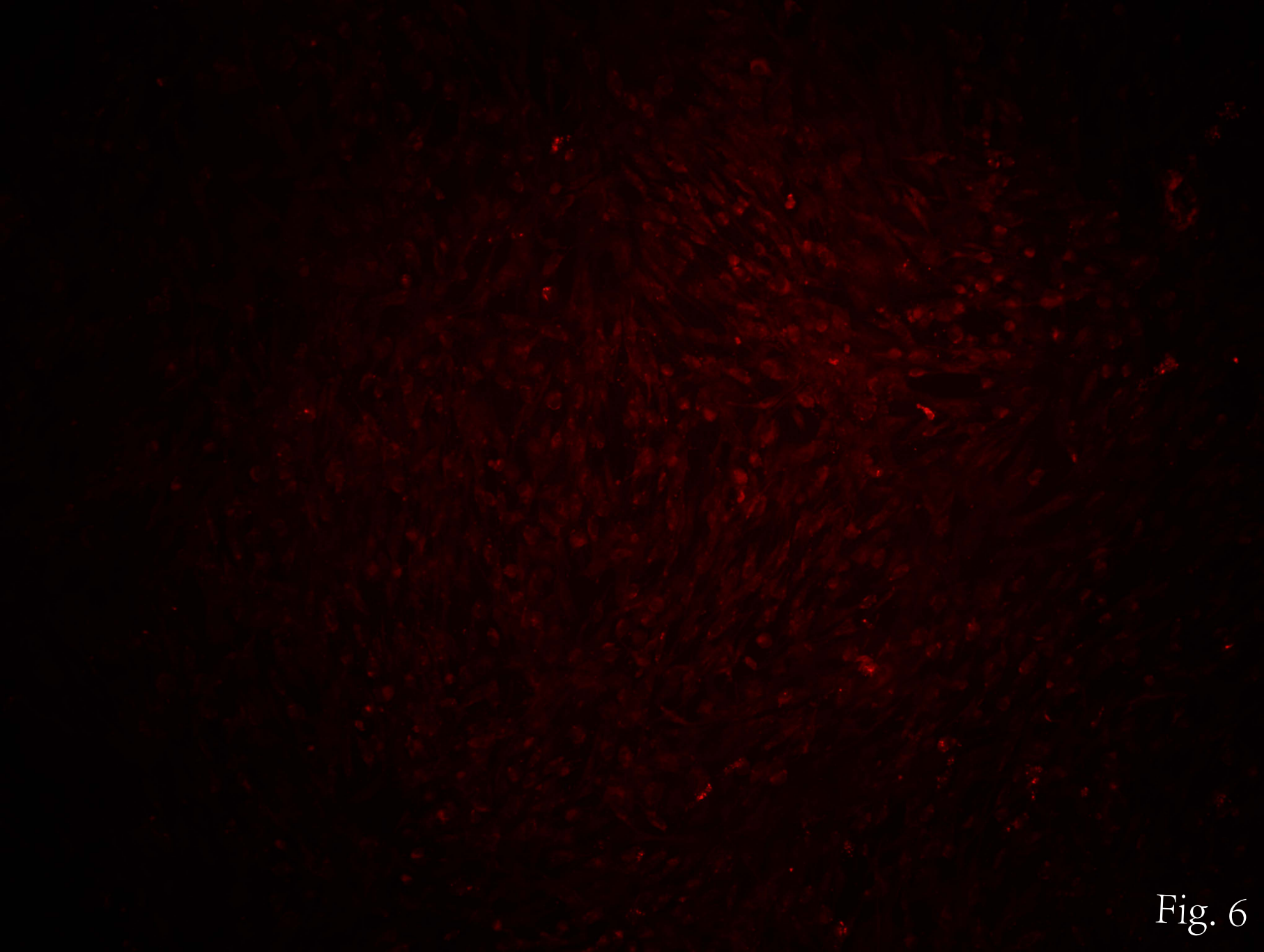

Fig. 6

Supplement: Supplementary file 1 — Additional file 1. [file 12917_2022_3529_MOESM1_ESM.pdf]
